# Development of an *Ex Vivo* Protocol to Model Bone Fracture in Laying Hens Resulting from Collisions

**DOI:** 10.1371/journal.pone.0066215

**Published:** 2013-06-13

**Authors:** Michael J. Toscano, Lindsay J. Wilkins, Georgina Millburn, Katherine Thorpe, John F. Tarlton

**Affiliations:** School of Veterinary Sciences, University of Bristol, Lower Langford, North Somerset, England; Ghent University, Belgium

## Abstract

Fractures of the keel bone, a bone extending ventrally from the sternum, are a serious health and welfare problem in free range laying hens. Recent findings suggest that a major cause of keel damage within extensive systems is collisions with internal housing structures, though investigative efforts have been hindered by difficulties in examining mechanisms and likely influencing factors at the moment of fracture. The objectives of this study were to develop an *ex vivo* impact protocol to model bone fracture in hens caused by collision, to assess impact and bird-related factors influencing fracture occurrence and severity, and to identify correlations of mechanical and structural properties between different skeletal sites. We induced keel bone fractures in euthanized hens using a drop-weight impact tester able to generate a range of impact energies, producing fractures that replicate those commonly found in commercial settings. The results demonstrated that impact energies of a similar order to those expected in normal housing were able to produce fractures, and that greater collision energies resulted in an increased likelihood of fractures and of greater severity. Relationships were also seen with keel’s lateral surface bone mineral density, and the peak reactive force (strength) at the base of the manubrial spine. Correlations were also identified between the keel and long bones with respect to both strength and bone mineral density. This is the first study able to relate impact and bone characteristics with keel bone fracture at the moment of collision. Greater understanding of these relationships will provide means to reduce levels of breakage and severity in commercial systems.

## Introduction

Egg production is currently undergoing dramatic changes in the housing of birds due to the 2012 ban of traditional battery cages in the EU (99/74/EC) and similar industry-guided, state-level movements in the United States. In the UK, there has been a dramatic increase in the use of extensive poultry layer systems, such as barn and free range, as an alternative to traditional caged systems, reaching 50% of production in 2011 [1] from 5% in 1980 [2]. Despite the multiple benefits a cage-free environment provides, such as greater ability to express natural behaviours, extensive systems introduce novel welfare problems. One of the most important of these welfare issues are keel bone fractures, a concern recognized by the United Kingdom’s Farm Animal Welfare Council [3]. The keel is a prominent bone extending ventrally from the sternum and serving as the point of attachment for the flight muscles [4]. Recent findings indicate that a major cause of keel damage within extensive systems is collisions with internal housing structures and descent from dedicated or informal perches where fracture prevalence correlated with the height of perches and slats [5]. These findings suggest descent from greater heights results in a greater kinetic energy at impact and increased risk of fracture. Perches in particular, though serving an obvious behavioural need, have been shown to increase fracture prevalence by 10–34% [5]. In contrast, relatively low perches (<77 cm above the ground [6]) or small inter-perch distances (<60 cm [7]) did not increase the frequency of keel fracture, indicating that birds are able to withstand impacts below a certain energy threshold. However, being unable to quantify factors likely to influence fracture risk at the moment of impact, such as kinetic energy, the development of interventions to reduce keel fractures has largely been a strategy of ‘hit-or-miss’. Thus, the principal objective of the current work was to develop a protocol to recreate keel bone fractures experimentally, and to permit precise quantification of various impact and bird-related factors likely to influence the likelihood of a fracture occurring. Factors were subsequently modelled mathematically against the collision outcome to gauge their relative importance and provide a methodology for data-driven solutions to reduce the frequency and severity of fractures. Additionally, mechanical and structural characteristics of the humerus and tibia were assessed to identify possible proxies for keel properties. These long bones were selected as their cylindrical shape allows for a broader range of calculations to be made (e.g., Young’s Modulus, Elastic and Plastic energies) allowing for a more comprehensive assessment of biomechanical properties. The bones, particularly the humerus, have a high incidence of fracture in commercial settings [8], have previously been used to assess heritability of bone strength [9], and thus are relevant to efforts investigating keel damage.

The objectives of this study were:

1. Development of an *ex vivo* keel impact protocol to experimentally create damage caused by collision to assess impact and bird-related factors (i.e. age, bone properties, breast muscling) affecting likelihood of keel fracture
2. Identification of correlations between the keel and the humeurs and tibia in terms of mechanical and structural properties.

## Materials and Methods

### Animals

All procedures were approved by the University of Bristol’s Animal Use Committee (University Identification Number: UB/12/027). Any and all data associated with the study can be provided by contacting the corresponding author. Hens were of the Lohman-Brown strain and collected from two commercial free range farms at three ages – 31(n = 40), 45(n = 40), and 65 (n = 55) wks of age. Individual birds were selected before range access was provided from various locations within the house to insure a stratified sample. Following collection, birds were brought to the University of Bristol’s School of Veterinary Science campus and killed within four hours by Euthatal injection (1 ml/bird,i.v.).

### Impact Testing Protocol

**Figure 1:**
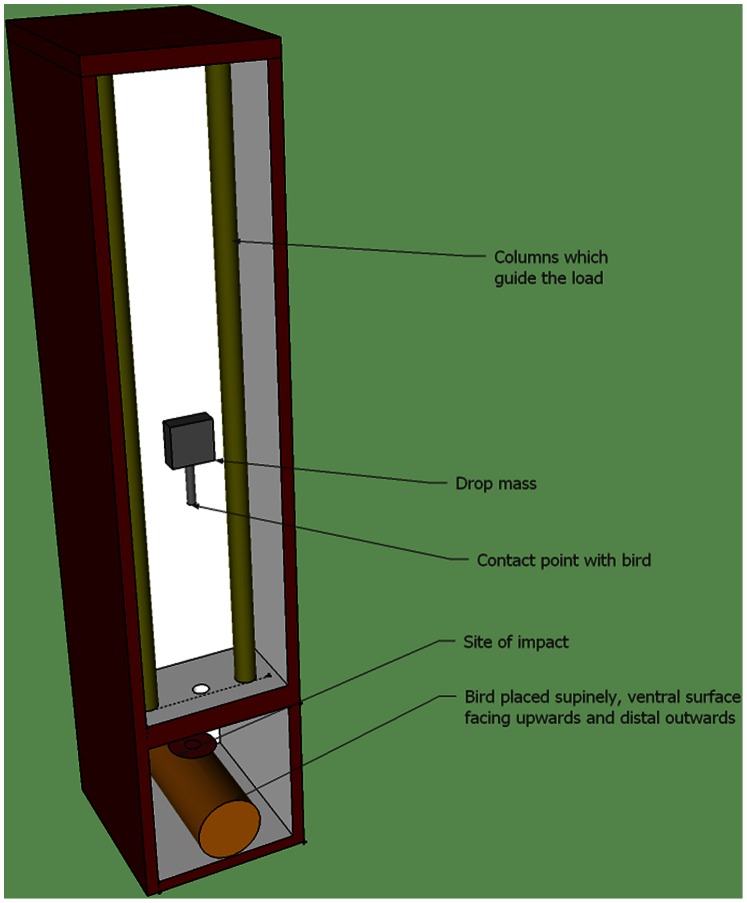
Schematic diagram of the impact device used to deliver loads during impacts. The device consisted of columns placed vertically between which an aluminium block (5×5×2 cm) of a specified mass (3.8 kg) could be dropped from specific heights onto a bird positioned at the base of the device. Runners affixed to the drop mass ensured the load could be delivered with high accuracy and precision to a target with minimum friction during travel. By altering the height from which the drop mass was released, the energy of impact could be changed accordingly. A rod extending from the base of the drop mass contained a force transducer which provided the peak force during impact. Actual contact with the bird was made with a crescent shaped metal flashing attached to the end of the rod.

**Figure 2:**
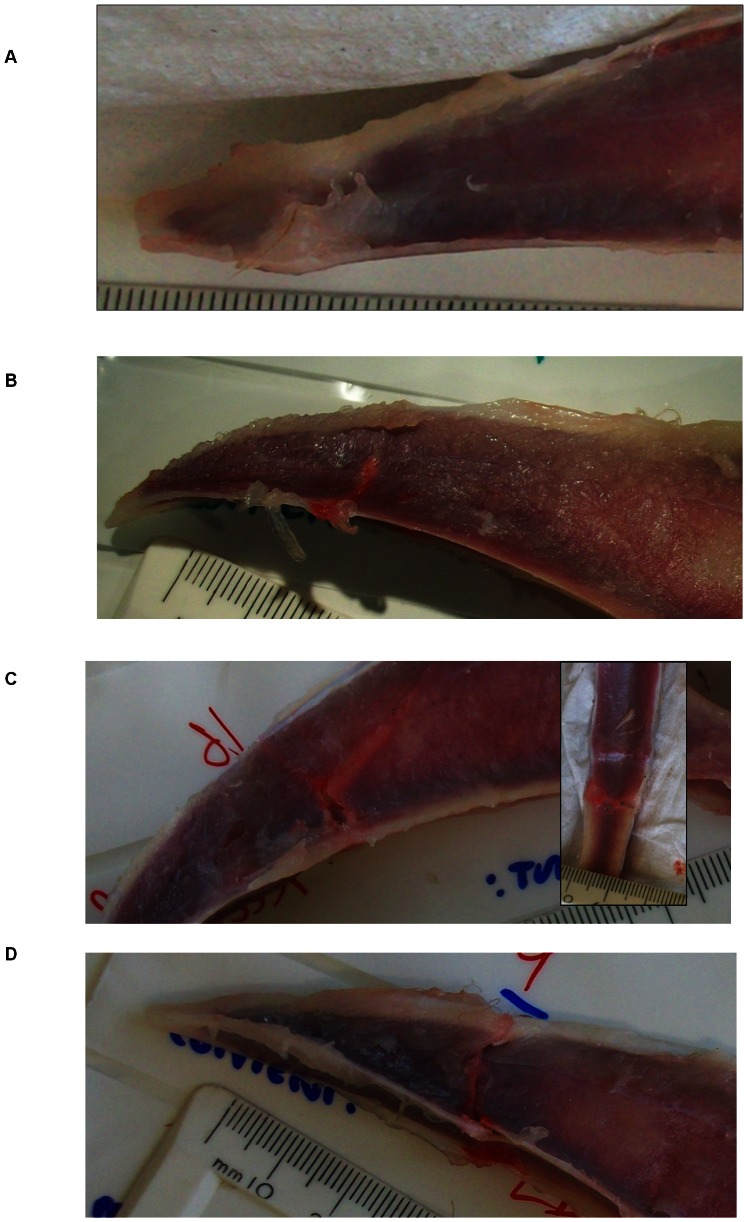
Severity score assessing the extent of damage. Hash marks indicate 1 mm.Panel A: Severity score = 0: No fracture. Panel B: Severity Score = 1: Small transverse fracture on the ventrolateral or dorsolateral aspect of the keel plate without extending to the ventral borders. Panel C: Severity Score = 2: Large transverse fracture extending from the ventral to the dorsal borders. The impact fracture can also be seen on the dorsoventral aspect of the base of the keel (inset). Panel D: Large transverse impact fracture extending from the ventral to the dorsal borders of the keel bone resulting in displacement of the tip of the keel bone.

Within 90 minutes of death, birds were weighed and placed into the base of a Rosand Drop-Weight Impact Tester (IFW Type 7) (Figure 1). Birds were positioned supinely such that the delivered load would make contact on the ventral surface (carina) of the keel approximately 2 cm above the most distal aspect. Thus, during the test procedure, the bird remained stationary while the drop mass was the moving component causing the collision. Although this would be the opposite of events leading to collisions, our assumption was that the energy involved in the collision is the critical factor. The drop mass was 3.8 kg and had a custom-designed, crescent-shaped impactor approximately 3 cm wide at its open point and 2.5 cm long. Based on previous trials, the impactor was designed to minimized shifting of the keel on contact. Impacts were delivered at one of five heights providing kinetic energies (E; KJ) from 42.6 to 70.9 KJ (based on the calculation of E = MV^2^ and V^2^ = 2AS, where V is velocity (m/s), M is mass of the load (Kg), S is drop height (m), and A is the acceleration due to gravity (9.8 m/s^2^). The impact tester calculated values for peak reactive force (KN) as measured by a strain gauge within the impact unit. Following impacts, birds were removed from the tester and the breast muscle assigned a 3-point categorical score of atrophy (poor muscling, standard muscling, full muscling). The measure of atrophy was included to provide a relatively robust indication of birds which had relatively poor muscling and thus exposed keels. We envisioned that if the measure was an effective predictor, it could be adopted by farm staff to provide an easily identifiable marker of susceptibility to keel damage. Following assignment of the atrophy score, the breast muscling was excised and weighed as a further measure of atrophy. Lastly, keels were carefully removed and examined for damage caused by the experimental impact. Record was made of whether a fracture occurred and, if so, given a severity score developed within our lab (Figure 2). Criteria used to grade keel damage severity were formulated following assessment in animals aged 65 wks, thus severity scores are only reported for 31 and 45 wks. Presence and severity of old breaks (breaks which had previously occurred and were in the process of healing) was also recorded using a scale previously reported [10].

**Figure 3:**
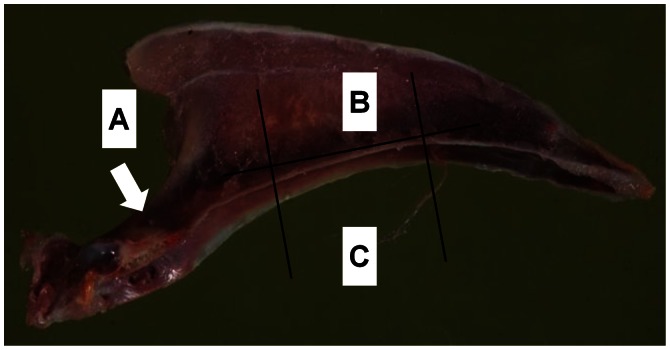
Areas of the keel for structural and biomechanical testing. Manubrial spine (A) for biomechanical testing where the arrow indicates the direction of applied force during biomechanical testing; lateral surface (B) and base (C) for bone mineral density quantification.

Following assessment, the midpoint of the keel in the cranial-caudal axis was identified and transverse cuts made approximately 1 cm on either side using a band saw to excise the center and cranial (manubrial spine) sections of the keel. A third coronal cut was made along the intramuscular line [4] separating the base and lateral surface (Figure 3). Lastly, the left humerus and tibia were removed. All bones were placed ‘as is’ in individual labelled plastic bags and stored at −20°C until subsequent analysis.

### Bone Mineral Density and Biomechanics

Quantification of bone mineral density was performed on humeri, tibiae and keels (base and surface) using dual energy X-ray absorptiometry (DEXA, Lunar PIXImus densitometer, Lunar Corp). Each bone was thawed to room temperature prior to testing, the length measured and midsection marked, and placed in the densitometer with the proximal surface parallel to the horizontal axis of the scan image. A 0.08 cm^2^ region was measured adjacent to the midsection line marked on the radiograph using a steel wire. Bone mineral density was subsequently calculated using the PIXImus software.

For biomechanical testing, bones were subjected to a 3-point breaking protocol previously reported [11] following bone mineral quantification. For the humeri and tibiae, bones were mounted on a mechanical testing frame across a supporting bridge with a gap of 4 cm and perpendicular load applied to the midpoint. The keel was positioned on a separate supporting bridge with a gap of 5 cm and support provided at the lateral portion of the keel’s most proximal aspect. The load was then applied to the base of the manubrial spine only, i.e, not the lateral surface where bone mineral density quantified (Figure 3). The load (N), displacement (mm), and total energy (J) required to reach structural failure were recorded.

### Statistical Modelling

#### *Ex vivo* fracture model

All data were assessed for normality and transformed as necessary. Continuous factors to be modeled were initially evaluated for multicolinearity by use of the variance inflation factor and tolerance options within SAS using Proc REG [12]. To assess the relationship of bird (age, bone biomechanical and mineral properties, breast muscling) and impact-related factors (peak force at collision, calculated kinetic energy of collision) with the likelihood of fracture, MlwiN [13] was used to model the probability of a fracture (response) occurring against the impact- and bird-related data (predictors) using a logit-link function where β_0_ represents the model intercept or overall reference. In this model, the occurrence of a fracture was considered as a binary response (fracture vs. no fracture). Breast muscle score and presence of an old break were classified as categorical variables while all other predictors were continuous. All continuous predictor variables (with the exception of age) were normalized to allow easier interpretation of model parameters.

A second analysis was conducted to assess the relationship between fracture severity and the predictors listed above using a subset of the data where fractures did occur (i.e., fracture scores of 1, 2, or 3). Statistical methods were similar in using a logit-link relationship to relate the response with predictors but employed a cumulative distribution model allowing all three categories to be modelled within a single model where the likelihood of a fracture score of ‘1 or 2’ or only ‘2’ occurring was compared against a score of ‘3’. Age was considered as a categorical factor as severity data from wk 65 was excluded.

For each analysis, the final model was created by generating a full model including all terms and 1st order interactions and then removing individual model components where comparison of the respective Z-ratio with a standard normal distribution was greater than 1.96 (p>0.05). Coefficients from the final model were used to generate odds ratios by exponentiating each coefficient. Odds ratios represent the risk of fracture for each of the specified variables; an odds ratio greater than one represents an increased risk of damage, whereas a value less than one represents a decreased risk.

#### Correlating different skeletal sites

To assess inter-bone correlations within birds, MlwiN was used to develop a mathematical relationship between keel bone properties and the equivalent measure in the tibiae or humeri. Specifically, for bone mineral density, values identified for the keel’s lateral surface and base were used, while for biomechanical properties, the base of the manubrial spine was used. For each, the value within the keel bone was modelled as the response with age, body mass, and the corresponding value in the long bone serving as predictors. Similar to methods in Objective 1, the final model was created by first generating a full model and then removing individual model components based on the associated Z-score. The percentage of variance in the keel response measure attributed to the long bone or age component of the model was also calculated by comparing variances of the respective models.

## Results

### *Ex vivo* Fracture Model

The frequency of fractures occurring appeared to increase with kinetic energy at collision where the highest percentage of the most severe fractures occurred at the greatest kinetic energy (i.e., 45% of collisions at 95.3 KJ resulting in a fracture score of ‘3’) (Table 1). The opposite pattern held for collisions with the least kinetic energy (i.e., 70% of collisions at 57.2 KJ resulted in a fracture score of ‘0’). Statistical modelling the likelihood of a fracture occurring (as a binary response, i.e., fracture vs. no fracture) identified the kinetic energy of impact, bone mineral density of the keel’s lateral surface, and peak reactive impact force to be effective predictors (Table 2). Increases in kinetic energy of impact increased the likelihood of a fracture occurring, whereas increasing bone mineral density and peak impact force decreased the likelihood of fracture. Specifically, an increase of one standard deviation in impact kinetic energy increased the risk of fracture by 3.6 times, while a similar increase in peak reactive force or bone mineral density of the keel surface approximately halved the risk of fracture. Alternatively, in terms of probabilities, assuming an increase of kinetic energy from 57 KJ to 68 K J (approximately 1 standard deviation) while other factors remain unchanged, the probability of a fracture occurring would rise from 52% to 80%. All other predictors, including age, were found to have no relationship with fracture incidence as a binary response.

**Table 1:** Outcome of collision events in terms of fracture and severity at tested impact kinetic energies.

Collision Energy (KJ)					
		57.2	71.1	82.6	95.3
0	n	14	12	8	5
	%	*70%*	*60%*	*40%*	*25%*
1	n	1	2	4	3
	%	*5%*	*10%*	*20%*	*15%*
2	n	4	3	4	3
	%	*20%*	*15%*	*20%*	*15%*
3	n	1	3	4	9
	%	*5%*	*15%*	*20%*	*45%*

Outcomes are listed under the column ‘Keel fracture score’ where ‘0’ would be the absence of fracture and ‘1’, ‘2’, and ‘3’ are fractures of increasing severity. For each kinetic energy, the actual number as well as percentage of collision events for each fracture score is provided.

**Table 2:** Model output and resulting odds ratios for the likelihood of a fracture occurring.

	Likelihood of fracture (binary)		
Term	Estimate	SE	Odds Ratio
β_o_	0.10	0.23	–
Impact Kinetic Energy (KJ)	1.28	0.34	3.60
Impact Peak Force (N)	−0.84	0.32	0.43
Keel Surface Bone Mineral Density (g/cm^3^)	−0.56	0.24	0.57

Fracture severity showed a relationship with keel surface bone mineral density and age that was uniform across severity scores (Table 3). Birds with greater keel surface bone mineral density or aged 45 wks (compared to 31 wks) had an increased likelihood of a less severe fracture occurring (‘1’ or ‘2’) compared to a ‘3’. Increased kinetic energy of impact associated with a reduced likelihood of a ‘2’ occurring in relation to a ‘3’, i.e. a ‘3’ was more likely to occur with increased kinetic energy.

**Table 3:** Model output for the associated likelihood of fractures occurring with varying severity.

	Fracture Severity Score				
	1 or 2		2		
Term	Estimate	SE	Estimate	SE	OR
β_o_	−2.76	0.76	−0.40	0.54	
Impact Kinetic Energy (KJ)	–		−0.95	0.49	0.39
Age† (45 wks)	2.13	0.84	2.13	0.84	8.43
Keel Surface Bone MineralDensity (g/cm^3^)	1.02	0.46	1.02	0.46	2.76

† For analysis of fracture severity, only data from 31 and 45 weeks was used, thus this term indicates the likelihood of fractures relative to 31 weeks.

The model used a cumulative distribution model that compared the likelihood of a ‘1 and 2’ or a ‘2’ occurring against a ‘3’. Calculated odds ratios are provided for the relevant outcome.

### Correlation across Skeletal Sites

Analysis of inter-bone correlations found bone mineral density of the keel base to correlate positively with that of the tibia and humerus where the inclusion of the long bone measures explained 10.0% and 13.4% of total keel base variation, respectively (Table 4). Age varied positively with the keel surface bone mineral density explaining 23.9% of total variation. Load at failure (peak reactive force) in the keel manubrial spine varied positively with load at failure in both the tibia and humerus where inclusion of the long bone measures explained 19.8% and 16.0% of variation in the keel, respectively (Table 5). Body mass correlated positively with keel displacement at failure explaining 6.9% of total variation; total energy at failure in the keel did not correlate to either predictor.

**Table 4:** Model output assessing inter-bone correlations within animals for bone mineral density.

	Constant		Age (Weeks)			Bone Effect		
Tibia	Estimate	SE	Estimate	SE	%Variance	Estimate	SE	%Variance
Keel Surface(g/cm^3^)¥	4.94	0.37	0.05	0.01	23.9%	NS (p > 0.05)		
Keel Base (g/cm^3^)	43.56	10.95	NS (p > 0.05)			0.12	0.04	10.0%
Humerus								
Keel Surface(g/cm^3^)¥	4.94	0.37	0.05	0.01	23.9%	NS (p > 0.05)		
Keel Base (g/cm^3^)	52.76	6.38	NS (p > 0.05)			0.11	0.03	13.4%

¥ Data was transformed using (response+1)∧.5

Model components detail the correlation between keel bone mineral density (g/cm^3^) with age and the associated measure in the tibia and humerus. The model components under ‘Bone Effect’ specify the correlation between the keel surface or base and corresponding measure in the tibia or humerus, e.g., changes in the keel base corresponded with a similar change of 0.12 in the tibia.

**Table 5:** Model output assessing inter-bone correlations within animals for biomechanics.

	Constant		Body Mass (Kg)			Bone Effect		
Tibia	Estimate	SE	Estimate	SE	%Variance	Estimate	SE	%Variance
Keel Load (N)	93.75	50.10	NS (p > 0.05)			0.85	0.20	19.8%
Keel Displacement (mm)	1.95	0.83	1.14	0.47	6.9%	NS (p > 0.05)		
Energy (J)	5.03	0.24	NS (p > 0.05)			NS (p > 0.05)		
Humerus								
Keel Load (N)	184.46	31.82	NS (p > 0.05)			0.53	0.14	16.0%
Keel Displacement (mm)	1.95	0.83	1.14	0.47	6.9%	NS (p > 0.05)		
Energy (J)	5.03	0.24	NS (p > 0.05)			NS (p > 0.05)		

Model components detailing the correlation between biomechanical properties at the base of the manubrial spine with age, and the associated biomechanical property in the tibia and humerus where the model components under ‘Bone Effect’ detail the correlation between the keel property and corresponding measure in the tibia or humerus, e.g., changes in the keel load corresponded with a similar change of 0.85 in the tibia.

## Discussion

Fractures to the keel of birds within extensive and caged systems is possibly the most critical welfare issue currently facing the egg production industry [3] given recent reports of the proportion of birds with keel fractures as high as 86% in some flocks within the UK [5]. Rates of fracture are likely to increase globally as the EU and US move away from traditional battery cage systems. Despite the severity of the problem, we have limited understanding of the factors that affect an individual bird’s susceptibility to keel damage. As a result of this lack of knowledge, attempts to reduce the occurrence and severity of keel damage are somewhat ‘hit-or-miss’ rather than objective and evidence-based. The current study sought to provide this information in an objective manner utilizing a novel, *ex-vivo* model of keel bone fracture where precisely controlled impacts were performed, allowing risk factors to be quantified at the time of collision, and related to fracture incidence and severity.

Experimental fractures resulting from our impact protocol appeared to follow the anticipated frequency pattern, in which greater energy of collision correlated to an increased likelihood of a fracture occurring and greater severity. Anatomically, experimental fractures resembled the physical patterns observed in naturally occurring breaks, e.g., low energy collisions resulting in a fractured base only (as with fractures characterized by a severity score of ‘1’) , whereas greater collision energies resulted in fractures extending into or entirely through the lateral surface. The notion that minor collisions would result in fractures characterized by breaks at the base alone is supported by our previously reported observations [14] of birds within caged systems (where collisions are likely to be of low energy). Thus, we believe our methodology effectively replicates fractures that would occur naturally within a commercial setting and offers the most appropriate means to assess keel bone damage given the described limitations. To confirm that our protocol recreates fractures that occur naturally, subsequent work will be required to validate our results; specifically, that the energy of collision predicted to cause fracture within given conditions results in a fracture of the expected anatomical nature.

While the anatomical appearance of fractures appeared to reflect the anticipated pattern, our methodology offered potential insight to clarify influencing factors affecting the frequency and severity of fractures. For instance, age appeared to not be a factor in the likelihood of a fracture occurring in conflict with previous observations that the proportion of birds with keel fractures increases with the age of birds [10], . The pattern of increased fractures with age is often attributed to bones weakened by gradual loss of structural bone over the course of the laying period and thus becoming osteoporotic with increasing age [16]. However, our data indicates that age did not influence fracture susceptibility, i.e., older birds were no more likely to experience a fracture than younger birds at similar collision energies. The lack of an age effect indicates that the reported increase in fractures with age relates to the accumulation of old breaks over time rather than greater susceptibility. Our model demonstrated increased bone mineral density of the keel surface associated with a reduced risk of fracture, a relationship in agreement with human studies [17], [18], and likely relates to the strength imparted by increased mineral density [18]. The role of bone strength in providing greater resistance to fracture is also supported by the inverse relationship between peak reactive force and fracture susceptibility. Our methods may provide an objective target value of bone mineral density and strength to reduce keel fractures utilizing breeding programs [19] and design of poultry diets [11].

Analysis of fracture severity also followed expectations in that less severe fractures were associated with reduced kinetic energy at impact and greater bone mineral density. In contrast to the binary response, age was shown to have a dramatic effect on the severity of keel damage. Of those birds with a fracture (‘1’, ‘2’ or ‘3’), hens aged 45 wks were eight times more likely to have a less severe fracture (‘1’ or ‘2’ vs. ‘3’) than birds of 31 wks. Taken together with the model of fracture occurrence discussed above, these results demonstrate that older birds were not necessarily more or less susceptible to developing fractures than younger birds; but, when fractures did occur, they were less severe. Given the relationship between increased keel surface bone mineral density and reduced fracture likelihood, this suggests that the greater bone mineral density of older birds may provide greater strength to reduce the severity, but not occurrence, of keel damage. Our biomechanical assessment of keel bone strength found no relationship to fracture likelihood, though the measure was a proxy as the force was applied at the base of the manubrial spine rather than where the actual impact was delivered (i.e., the lateral surface) and thus may not be representative.

Several relationships were identified between the keel and tibiae and humeri in terms of bone mineral density and load at failure. Surprisingly, our analysis indicated that while peak load of the keel at failure correlated with those of both the tibiae and humeri, it was the tibiae that exhibited the stronger association. Our expectation was that the humeri, which share several opposing muscle attachments with the keel (in particular the pectoralis) [4], and therefore similarly exposed to load during muscle contractions, would have a greater correlation to the keel than the tibiae. Bishop *et al.* [9] reported keel radiographic density, which could be taken as an indication of strength, to have a greater correlation with biomechanically tested strength of the tibia in comparison to the humerus in end of lay hens, thus our findings do not to appear to be an artefact. Possible explanations for this are that the keel bone is loaded during bipedal locomotion, or that the short bursts of flight seen in commercial poultry houses (more like jumping, though the wings are extended) exact a load on the tibiae during landing. Alternatively, the manubrial spine which would not be directly exposed to the forces during either leg or wing motion, may not be the most appropriate site for mechanical testing. The site was chosen as it is normally free of damage and thus offers an area where loading can be applied consistently across samples. Future work would benefit from development of a protocol that assesses mechanics in a manner that reflects typical biological loading, e.g. the ventral or lateral surface.

### Conclusion

The current work is the first report of an *ex vivo* protocol for inducing keel fractures in laying hens that is able to replicate anatomical damage found in commercial settings. The protocol allows us to relate energy of an individual collision with likelihood of fracture occurrence and severity and mathematically model the contribution of bird factors such as age or bone strength from which we can identify the most effective targets for prevention of keel damage. The outcomes followed the expected pattern where lesser collision energies resulted in a decreased likelihood of fractures overall as well as being less severe, and greater bone strength and mineral density, but not age, also mitigated fractures. Correlations were found between measurements of the keel and long bones that suggest that the latter could be used as proxies for keel properties, though further work may be warranted to identify a more biologically relevant area than the manubrial spine for biomechanical testing.
